# Differences in systemic adaptive immunity contribute to the ‘frequent exacerbator’ COPD phenotype

**DOI:** 10.1186/s12931-016-0456-y

**Published:** 2016-10-28

**Authors:** Jasper X. Geerdink, Sami O. Simons, Rebecca Pike, Hans J. Stauss, Yvonne F. Heijdra, John R. Hurst

**Affiliations:** 1 Department of Respiratory Medicine, Radboud University Medical Centre, Nijmegen, The Netherlands; 2 UCL Respiratory, University College London, London, UK; 3 Institute of Immunity and Transplantation, University College London, London, UK

**Keywords:** COPD, COPD exacerbation, Respiratory immunology, Flow cytometry, Adaptive immunity

## Abstract

**Background:**

Some COPD patients are more susceptible to exacerbations than others. Mechanisms underlying these differences in susceptibility are not well understood. We hypothesized that altered cell mediated immune responses may underlie a propensity to suffer from frequent exacerbations in COPD.

**Methods:**

Peripheral blood mononuclear cells (PBMCs) were obtained from 24 stable COPD patients, eight frequent exacerbators (≥3 diary-card exacerbations/year) and 16 infrequent exacerbators (< 3 diary-card exacerbations/year). Detailed multi-parameter flow cytometry was used to study differences in innate and adaptive systemic immune function between frequent and infrequently exacerbating COPD patients.

**Results:**

The 24 COPD patients had a mean (SD) age of 76.3 (9.4) years and FEV_1_ 1.43 (0.60)L, 53.3 (18.3)% predicted. PBMCs of frequent exacerbators (FE) contained lower frequencies of CD4+ T central memory cells (CD4+ Tcm) compared to infrequent exacerbators (IE) (FE = 18.7 %; IE = 23.9 %; *p* = 0.035). This observation was also apparent in absolute numbers of CD4+ Tcm cells (FE = 0.17 × 10^6/mL; IE = 0.25 × 10^6/mL; *p* = 0.035). PBMCs of FE contained a lower frequency of CD8+ T effector memory cells expressing HLA-DR (Human Leukocyte Antigen - D Related) compared to IE COPD patients (FE = 22.7 %; IE = 31.5 %; *p* = 0.007).

**Conclusion:**

Differences in the adaptive systemic immune system might associate with exacerbation susceptibility in the ‘frequent exacerbator’ COPD phenotype. These differences include fewer CD4+ T central memory cells and CD8+ T effector memory cells.

**Trial registration:**

Not applicable.

## Background

Patients with chronic obstructive pulmonary disease (COPD) are prone to periodic deteriorations in respiratory health called exacerbations. Exacerbations impose a huge burden on patients by reducing quality of life [1], accelerating lung function decline [2] and increasing the risk of death [3]. Exacerbations also pose a significant financial burden on health care systems due to supplementary treatment and increased hospital admissions [4, 5].

Most exacerbations are caused by infections with respiratory viruses and or alterations in the lung bacterial flora termed dysbiosis. Mechanisms underlying an increased susceptibility to infections are not well understood. Key immune effector cells are abundant in the lungs of COPD patients, and data have shown that they may fail to launch an effective immune response when encountering an infectious pathogen [6]. It is has been suggested that a deficient immune response to pathogens may be attributed to decreased effector cell function and an increased number of suppressive T-regulatory cells [7]. In addition, Kalathil [8] reported an increased number of exhausted effector T-cells, characterised by expression of PD-1 (marker for programmed cell death and cell exhaustion), in the systemic circulation of patients with COPD compared to healthy controls. Similarly, McKendry [9] found increased PD-1 expression in CD8+ cells in lung tissue samples when comparing COPD patients to healthy controls. These changes in the immune system in COPD may lead to an immune paralytic state and thus predispose to recurrent infections [7].

Although this hypothesis is attractive, translational studies correlating changes in systemic immune function with exacerbation frequency in COPD are lacking. In particular, it is not known if such changes are seen in the ‘frequent exacerbator’ exacerbation-susceptibility phenotype [10]. The aim of the present study was to investigate whether alterations in the cell mediated immune system might underlie the frequent exacerbator phenotype in COPD.

## Methods

### Study population

This analysis comprises data from a subset of stable COPD patients from the London COPD cohort, collected between May 2012 and April 2013. The setting, recruitment and monitoring of the London COPD cohort have been described previously [1, 2, 11–16]. In short, patients with moderate to severe COPD were included. Patients were trained to record daily any worsening of respiratory symptoms on diary cards and were subsequently followed for 1 year to record exacerbations of COPD.

COPD was defined as a post-bronchodilator Forced Exhaled Volume in 1 s (FEV_1_) < 80 % predicted, a FEV_1_/FVC ratio <70 %, and β2-agonist reversibility <12 % and/or 200 ml. Patients with asthma were excluded. A COPD exacerbation was defined as an increase of two major symptoms (increased breathlessness, sputum volume or purulence) or one major and one minor symptom (cold, increased cough, increased wheeze, sore throat). Patients were categorised as frequent (FE) or infrequent (IE) exacerbators defined as three or more versus two or fewer diary card (symptom based) exacerbations per year, respectively. We used three exacerbations for defining a frequent exacerbator (FE) as we included both treated and untreated exacerbations.

### Blood samples

Blood samples from 24 stable COPD patients were collected during the study period. Peripheral blood mononuclear cells (PBMCs) were obtained from these blood samples by means of density centrifugation and stored at −180 °C in vapour-phase nitrogen in the UCL-RFH Biobank prior to this analysis.

For thawing, PBMCs were re-suspended in a 30 mL solution of pre-warmed (37 °C) RPMI-1640, Heat-Inactivated Foetal Bovine Serum, Penicillin, Streptomycin, and L-Glutamine (R20). The R20 containing the PBMCs was then centrifuged for 5 min at 1500 rpm at 20 °C. Once centrifuged, the supernatant was removed and the remaining pellets were re-suspended in 10 mL of phosphate buffered saline (PBS). Next, viable cell counts were determined by the trypan blue exclusion (Neubauer hemocytometer). The cell concentration was adjusted to 10 × 10^6^/mL in supplemented PBS with additional 1 % (volume/volume) foetal calf serum (FCS); 1 × 10^6^ cells were dispensed into a 96 well plate for antibody labelling.

### Antibody labelling

We used a detailed immune screening panel developed at The Royal Free Institute of Immunity and Transplantation, specifically designed for studying infection susceptibility and transplant rejection [17]. 1 × 10^6^ PBMCs were stained for a T-cell panel, an innate panel, and isotype controls. Before antibody labelling, the suspensions were incubated with purified human IgG (Sigma) to block Fc receptors, reducing non-specific antibody binding.

The T-cell panel aliquots were incubated with combinations of CD3-*PECy7* (clone SK7), CD4-*v500* (clone RPA-T4), CD8-*v450* (clone RPA-T8), CD45R0-*PECF594* (clone UCHL1), CD62L-*APC* (clone DREG-56), CD25-*APC Cy7* (clone M-A251), CD127-*FITC* (clone HIL-7R-M21) (all from BD Biosciences), HLA-DR-*PerCPCy5.5* (clone LN3) (eBioscience), and CD279-*PE* (clone EH12.2H7) (Biolegend).

The innate panel aliquots were incubated with the following combination of antibodies: CD3-*PECy7* (clone SK7), CD4-*v500* (clone RPA-T4), CD8-*v450* (clone RPA-T8), CD45R0-*PECF594* (clone UCHL1), CD56-*APC* (clone NCAM16.2), CD16-*APC H7* (clone 3G8), iNKT-*PE* (clone 6B11), and Vd2-*FITC* (B6) (all from BD biosciences).

For each sample in the T-cell panel an individual isotype control was used. The isotype controls were labelled with CD3-*PECy7* (clone SK7), CD4-*v500* (clone RPA-T4), CD8-*v450* (clone RPA-T8), CD45R0-*PECF594* (clone UCHL1), IgG1k-*APC Cy7* (clone MOPC-21) (all from BD biosciences), IgG1k-*APC* (clone MOPC-21), IgG1k-*FITC* (clone MOPC-21), IgG1k-*PE* (clone MOPC-21) (Biolegend), and IgG2b-*PerCPCy5.5* (clone N/S) (eBioscience).

### Flow cytometry

Flow cytometry analyses were performed with a four-laser SORP (special order research product) BD LSRFortessa™ cytometer using the BD FACSDiva™ software V.6.0.1. The results were analysed using FlowJo X 10.0.7r2 software.

### Statistical analysis

Cell populations in frequent exacerbators were compared with infrequent exacerbators. Flow cytometry results were expressed as a percentage of parent cell and total number of cells calculated from the total lymphocyte count. Statistical analyses were performed using SPSS (IBM SPSS Statistics, Version 22.0. Armonk, NY: IBM Corp). Differences in percentages between groups were compared using Fisher’s exact test and differences in total number of cells using the Mann Whitney U test. A two-tailed p value of < 0.05 was considered to indicate significance. We did not correct for multiple testing because we further analysed our data with principal component analysis (PCA) and statistical multiplicity does not affect PCA. The PCA is an unsupervised technique that is used to project high-dimensional data into a new co-ordinate system. The projection of data into a new co-ordinate system is performed to find meaningful structure within extensive data sets; thereby deriving parameters, so-called principal components, that best describe the variance in an entire data set. The effectiveness of this analysis can be quantified by calculating the relative amount of variation that each principle component describes (expressed as a percentage of the total variance).

We performed a stepwise multivariable linear regression analysis with CD4+ memory T-cells as independent variable and age (in years), smoking status (in pack-years), severity of COPD (% FEV predicted) as dependent variables to identify which clinical parameters associate with central memory T-cells.

Research ethics approval was obtained from the Research Ethics Committee of the Royal Free Hampstead NHS Trust where this work was undertaken (reference number 05/Q0501/126). All subjects provided written informed consent.

## Results

Twenty-four patients participated in this study (eight frequent and 16 infrequent exacerbators). The demographic and lung function data are presented in Table 1 *.* The mean (SD) age of the patients was 76.3 (9.4) years and FEV_1_ 53.3 (18.3) % predicted. There were no statistically significant differences between the frequent and infrequent groups in age, gender, lung function parameters, or smoking history.

**Table 1:** Study population

Characteristics	Frequent exacerbation (*n* = 8)	Infrequent exacerbation (*n* = 16)	p
Age (years)	80.6 ± 11.2	74.1 ± 8.0	0.169
Gender (male)	6	11	0.751
Exacerbation Frequency (/y)	3.82 ± 0.92	1.70 ± 0.81	NA
Smoking Status (ex-smoker)	7	10	0.204
Pack years	51.6 ± 30.4	44.9 ± 26.7	0.605
FEV1 (L)	1.21 ± 0.47	1.54 ± 0.64	0.163
FVC (L)	2.41 ± 0.58	2.96 ± 0.86	0.079
FEV1 %pred (%)	52.6 ± 19.4	53.7 ± 18.4	0.892
FVC %pred (%)	91.1 ± 32.7	82.9 ± 18.7	0.527
FEV1/FVC	0.52 ± 0.20	0.51 ± 0.15	0.928

Data expressed as mean ± SD except absolute number for gender and smoking status. Significance was calculated with chi-square or t test as appropriate. *FEV*_*1*_ forced expiratory volume in 1 s, *FVC* forced vital capacity, *%pred* the percentage of predicted

### Susceptibility to exacerbation

**Fig. 1:**
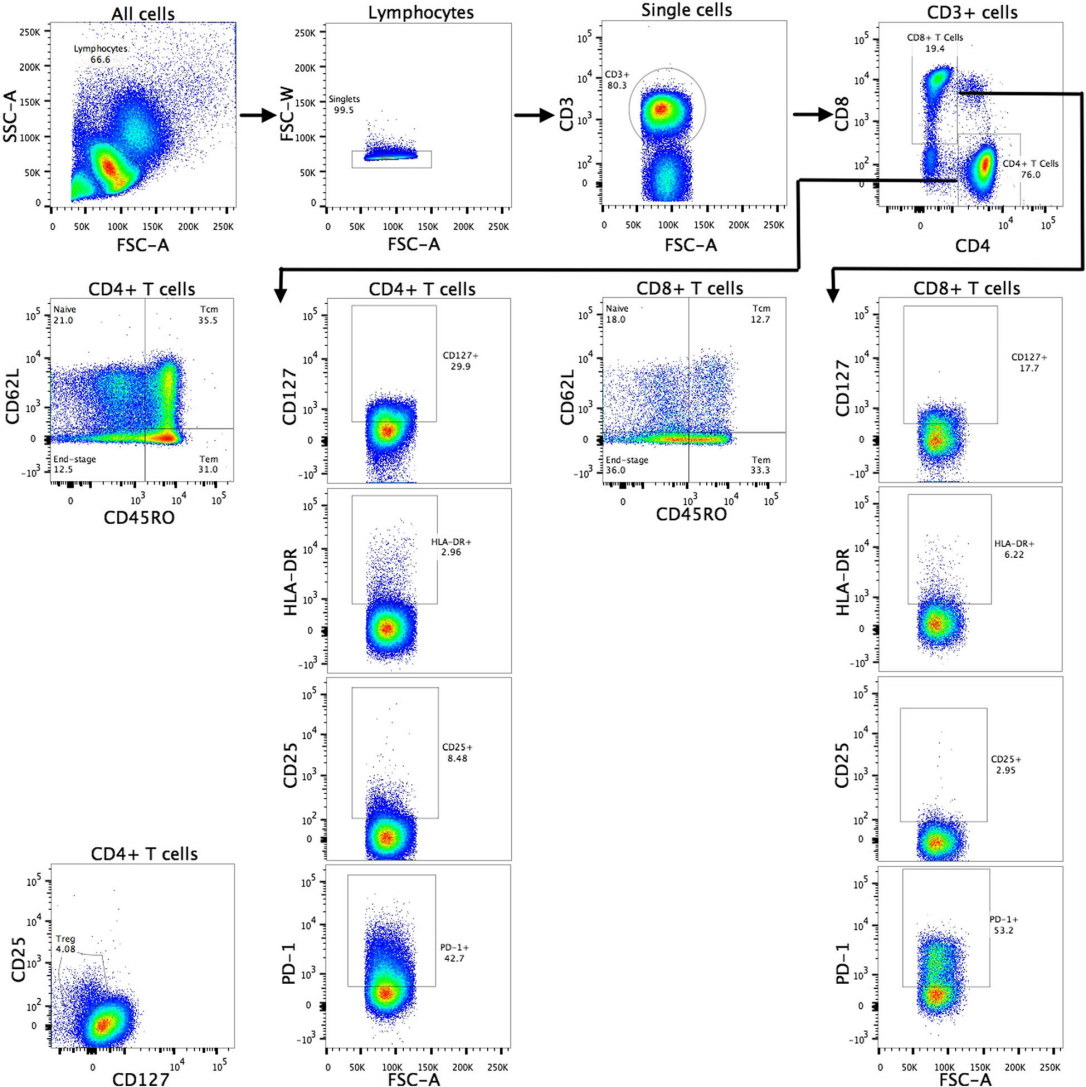
Gating strategy for adaptive immune cells. Lymphocytes were gated based on their characteristic scatter patterns. Lymphocytes were then classified based on CD3 expression to identify T cells, which were divided into CD4^+^ and CD8^+^ T cells, prior to separation into their four main subsets of naive, central memory (Tcm), effector memory (Tem), and end-stage T cells (Tend); this separation was based on the expression of CD45R0 and CD62L. Tcm cells: CD45R0+/CD62L+, Tem cells: CD45R0+/CD62-, Naive T cells: CD45R0-/CD62L+, and Tend cells: CD45R0-/CD62L-. The CD4+ and CD8+ T cells were subsequently analysed (including each of the main subsets) for expression of the markers CD127, HLA-DR, CD25 and PD-1. Regulatory T-cells were identified based on the high expression of CD25 and low expression of CD127 in CD4+ T cells

**Fig. 2:**
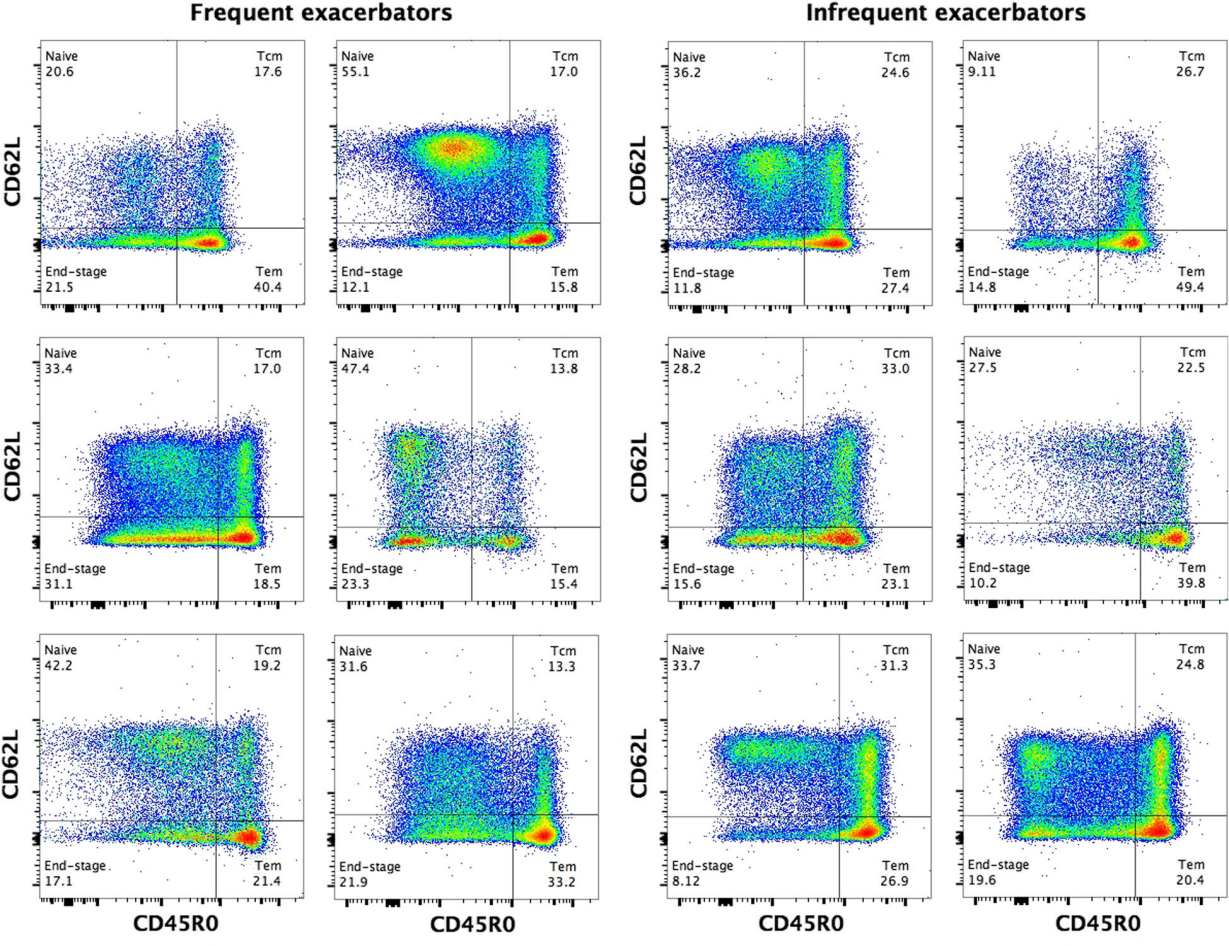
Representative plots of CD4+ T central memory cells in patients with frequent exacerbations and in patients who exacerbate infrequently. CD4+ Tcm cells were identified as CD45R0+ and CD62L+ (*top-right gate*). The left plots present the memory profile belonging to a COPD patient susceptible to frequent exacerbations and the right plots are from patients who exacerbate infrequently. Tem = CD4+ T effector memory (*bottom-right gate*)

**Fig. 3:**
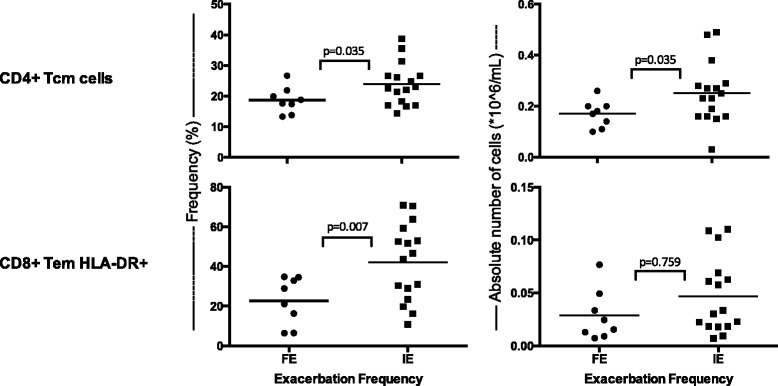
Susceptibility to Exacerbation in COPD is associated with reduced CD4+ Tcm and CD8+ Tem HLA-DR+. Graphs show the frequency (*left*) and absolute number (*right*) of CD4+ Tcm cells in frequent exacerbators (FE) and infrequent exacerbators (IE). Horizontal lines indicate the mean. Each dot represents an individual patient

The flow cytometry results by exacerbation frequency are presented in Tables 2 and 3 with the gating illustrated as Fig. 1 *.* Susceptibility to exacerbation was associated with differences in acquired, but not innate immune cells. Figure 2 depicts five representative plots of the division of CD4+ T cells into memory subsets. Frequent exacerbators had a lower frequency of CD4+ central memory T cells (CD4+ Tcm) compared to infrequent exacerbators (FE =18.7 %, IE =23.9 %, *p* = 0.035) (Fig. 3). There was also a decrease in the absolute number of CD4+ Tcm cells between these groups (FE =0.170 × 10^6^/mL, IE =0.250 × 10^6^/mL, *p* = 0.035). In addition, we also found a lower percentage of activated CD8+ T effector memory cells (CD8+ Tem HLA-DR) in the frequent exacerbator group compared to the infrequent exacerbators (FE =22.7 %, IE =31.5 % *p* = 0.007, Fig. 3). HLA-DR is a marker for early activation of T cells. The absolute number of these cells (FE = 0.020 × 10^6^/mL, IE = 0.032 × 10^6^/mL *p* = 0.759) was not statistically different. We found no statistically significant differences in other subsets of adaptive immune cells, including regulatory T cells (FE = 6.6 %, IE = 7.6 %, *p* = 0.270). The expression of PD-1, a marker which is expressed when cells are exhausted and/or will undergo apoptosis, did not significantly differ in various adaptive immune cells.

**Table 2:** Flow cytometry frequency results from COPD patients susceptible to frequent vs. infrequent exacerbations

Cell type	FE (*n* = 8) Mean ± SD % / Median (IQR) %	IE (*n* = 16) Mean freq. ± SD % / Median (IQR) %	p
CD3+	66.7 ± 12.0	77.2 ± 10.7	0.291
CD4+	71.2 ± 17.0	73.6 ± 14.7	0.748
CD4+ HLA-DR+	5.2 (2.3–7.0)	2.8 (2.3–4.9)	0.417
CD4+ PD-1+	29.8 ± 9.2	27.8 ± 11.5	0.652
CD4+ Naive	37.5 ± 13.7	34.8 ± 13.3	0.651
CD4+ Tcm	18.7 ± 4.3	23.9 ± 6.9	0.035
CD4+ Tem	25.5 ± 11.1	25.6 ± 11.3	0.983
CD4+ Tem PD-1+	60.2 ± 7.6	51.6 ± 13.5	0.059
CD4+ End	18.3 ± 4.9	15.8 ± 6.4	0.287
CD8+	21.0 (12.6–31.3)	19.2 (12.5–25.8)	0.787
CD8+ HLA-DR+	12.4 (4.2–23.4)	8.7 (6.4–20.9)	0.928
CD8+ PD-1+	37.9 ± 11.1	36.6 ± 13.4	0.800
CD8+ Naïve	11.1 ± 6.5	14.3 ± 7.8	0.294
CD8+ Tcm	15.0 (6.7–17.5)	13.0 (10.9–18.8)	0.834
CD8+ Tem	43.9 ± 15.5	37.4 ± 8.6	0.300
CD8+ Tem HLA-DR+	22.7 ± 13.5	31.5 ± 13.0	0.007
CD8+ End	27.4 ± 13.5	31.5 ± 13.0	0.488
Treg	6.6 ± 2.0	7.6 ± 2.0	0.270
Treg PD-1+	39.2 ± 14.0	35.3 ± 12.7	0.521
NK	62.9 ± 18.8	57.0 ± 17.6	0.473
NK CD56^bright^	2.8 ± 2.1	3.5 ± 2.2	0.594
NK CD56^dim^	58.8 ± 19.8	53.4 ± 18.4	0.530
NK CD56^bright^CD16^dim^	1.8 (1.1–4.8)	2.5 (1.4–4.4)	0.697
NK CD56^dim^CD16^bright^	48.1 ± 20.3	45.9 ± 18.7	0.803
NK CD56 + CD56^bright^	3.5 (1.6–8.9)	6.5 (2.3–9.1)	0.358
NK CD56 + CD56^dim^	92.7 (87.9–98.3)	93.4 (90.6–97.6)	0.903
iNKT	1.6 (1.0–3.3)	1.2 (0.73–2.5)	0.417
γδ T	0.78 (0.34–1.4)	0.32 (0.17–0.70)	0.153
γδ T CD56+	30.9 ± 15.4	32.3 ± 14.7	0.834
CD4+ : CD8+ ratio	45 ± 8.0	45 ± 6.2	0.959

Table shows the mean frequency (%) of cells based on their parent cell line. Significance was calculated using t test or Mann-Whitney U test as appropriate. *PD-1+* cell marker for programmed cell death, *End* end-stage T cell, *Treg* regulatory T cell, *NK* natural killer, *iNKT* invariant natural killer T cell

**Table 3:** Flow cytometry absolute number of cells results from COPD patients susceptible to frequent vs. infrequent exacerbations

Cell type	FE (*n* = 8) Mean abs No. ± SD/Median abs No. (IQR) *10^6/ml	IE (*n* = 16) Mean abs No. ± SD/Median abs No. (IQR) *10^6/ml	p
CD3+	1.33 ± 0.38	1.42 ± 0.53	0.665
CD4+	0.91 ± 0.19	1.07 ± 0.42	0.223
CD4+ HLA-DR+	0.05 (0.02–0.07)	0.03 (0.02–0.04)	0.490
CD4+ PD-1+	0.27 ± 0.09	0.28 ± 0.14	0.785
CD4+ Naive	0.34 ± 0.12	0.38 ± 0.21	0.505
CD4+ Tcm	0.17 ± 0.05	0.25 ± 0.12	0.035
CD4+ Tem	0.23 ± 0.12	0.26 ± 0.14	0.669
CD4+ Tem PD-1+	0.14 ± 0.07	0.13 ± 0.07	0.666
CD4+ End	0.17 ± 0.06	0.17 ± 0.11	0.826
CD8+	0.28 (0.13–53)	0.30 (0.13–0.40)	0.697
CD8+ HLA-DR+	0.05 (0.01–0.09)	0.03 (0.01–0.06)	0.653
CD8+ PD-1+	0.12 ± 0.09	0.11 ± 0.07	0.707
CD8+ Naïve	0.04 ± 0.02	0.04 ± 0.03	0.662
CD8+ Tcm	0.05 (0.02–0.06)	0.04 (0.02–0.07)	0.976
CD8+ Tem	0.16 ± 0.12	0.11 ± 0.07	0.368
CD8+ Tem HLA-DR+	0.02 ± 0.01	0.03 ± 0.06	0.759
CD8+ End	0.13 ± 0.05	0.09 ± 0.06	0.575
Treg	0.06 ± 0.03	0.08 ± 0.04	0.173
Treg PD-1+	0.02 ± 0.01	0.03 ± 0.01	0.296
NK	0.51 ± 0.24	0.30 ± 0.17	0.336
NK CD56^bright^	0.01 ± <0.01	0.01 ± <0.01	0.572
NK CD56^dim^	0.39 ± 0.12	0.18 ± 0.14	0.373
NK CD56^bright^CD16^dim^	0.01 (<0.01–0.02)	0.01 (<0.01–0.01)	0.490
NK CD56^dim^CD16^bright^	0.35 ± 0.14	0.15 ± 0.13	0.398
NK CD56 + CD56^bright^	0.02 (0.01–0.03)	0.01 (<0.01–0.02)	0.653
NK CD56 + CD56^dim^	0.29 (0.21–0.49)	0.26 (0.12 0.41)	0.490
iNKT	0.02 (0.01–0.03)	0.01 (0.01–0.03)	0.214
γδ T	0.01 (<0.01–0.02)	<0.01 (<0.01–0.01)	0.153
γδ T CD56+	0.01 ± 0.01	0.01 ± 0.01	0.544

Table shows the absolute number of cells in 1 ml of peripheral blood. Significance was calculated using t test or Mann-Whitney U test as appropriate. *Abs No.* absolute number, *PD-1+* cell marker for programmed cell death, *End* end-stage T cell, *Treg* regulatory T cell, *NK* natural killer, *iNKT* invariant natural killer T cell

We did not define differences in innate immunity between FE and IE. For example, the proportion of natural Killer (NK) cells (FE = 62.9 %, IE = 57.0 %, *p* = 0.473) and the variety of NK cell subsets did not differ significantly by susceptibility to exacerbation.

#### Principal Component Analysis (PCA)

**Fig. 4:**
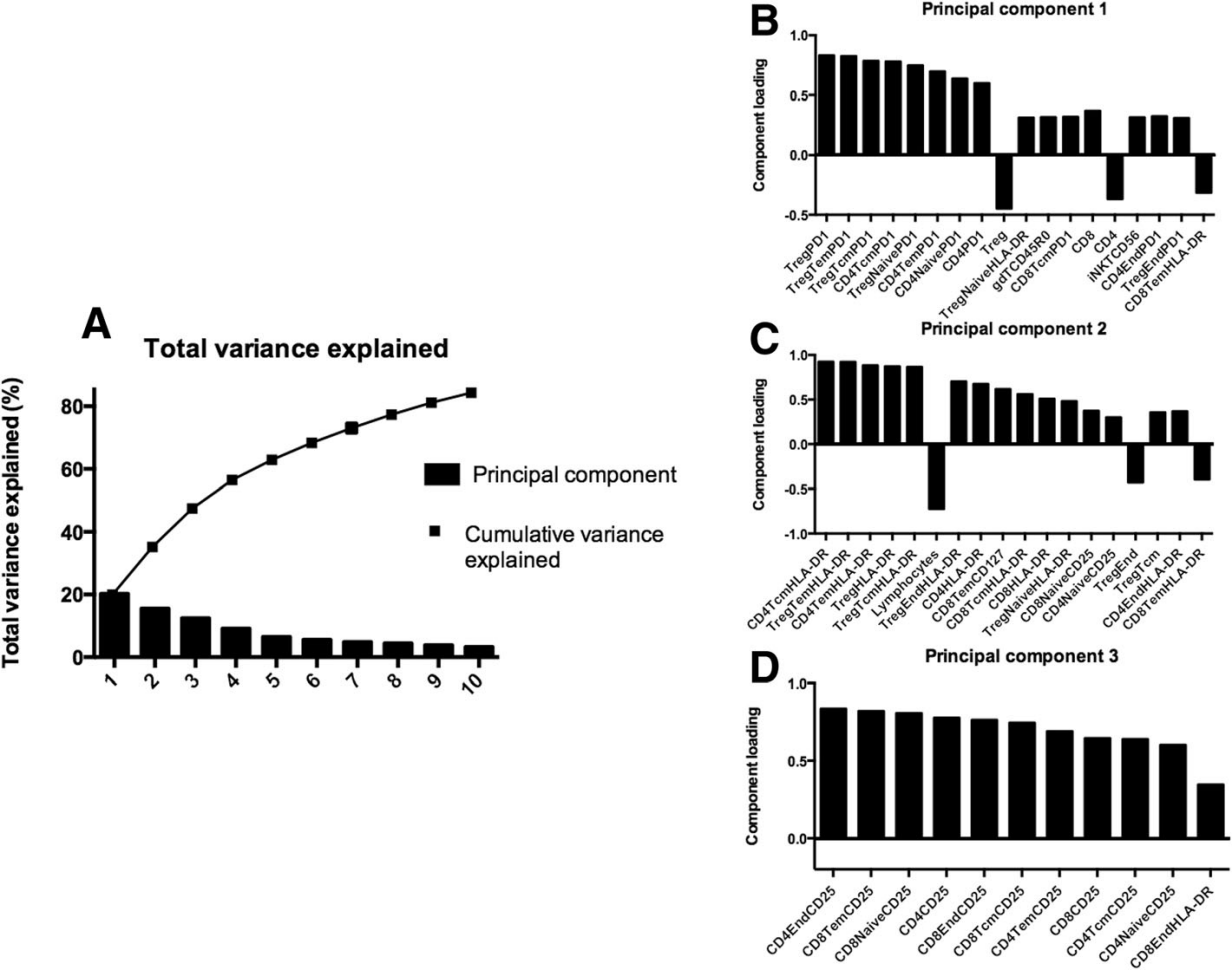
Principal component analysis. Graph presenting the results of the principal component analysis; this analysis was unable to concretely stratify patients based on frequency of exacerbations, which suggests high heterogeneity. **a** Graph presenting the percentage of variance explained, bars represent each separate principal component (PC), the line represents the cumulative percentage of these PCs. **b-d** The bar graphs present the principle component analysis parameter loadings for the first three PCs. The bars illustrate the weighting coefficients, which demonstrates the contribution each component (in this case: cell types) has in relation to their respective PC

Data were further analysed using an unsupervised PCA. This demonstrated that the first principal component (PC) explained 20 % of the total variance. Adding a second and a third covered 36 % and 48 % respectively. Adding more PCs explained additional variance, however, the contribution from further PCs was limited (Fig. 4a). The principle component analysis parameter loadings (weighting coefficients) for the first PC are plotted in Fig. 4b-d. The first PC associated most closely with cells expressing PD-1, and there had been a trend to a statistically lower proportion of CD4+ Tem cells expressing PD-1 in frequent versus infrequent exacerbators (Table 2). The second PC associated most closely with CD4+ Tcm. However, the PCA was unable to differentiate frequent from infrequent exacerbators and variance in the dataset reflects high heterogeneity between COPD patients in immunological profiles. This, in itself, is an important observation.

#### Multiple linear regression analysis

To investigate which clinical characteristics were correlated with CD4+ central memory cells, we conducted a multiple linear regression analysis (Table 4). This analysis shows that the only factor significantly associated with CD4+ central memory T-cells was exacerbation frequency.

**Table 4:** Stepwise multiple linear regression model investigating clinical variables associated with CD4+ central memory T-cells

	B (SE)	p
Exacerbation frequency	−2.62 (1.04)	0.03
FEV_1_ % predicted	0.42 (0.16)	0.70
Smoking history (pack years)	0.38 (0.07)	0.10
Age	0.12 (0.02)	0.68

B-value indicates the individual contribution of each predictor to the model. All parameters were included in the multiple linear regression using the stepwise method

## Discussion

We have shown that alterations in the cell mediated immune system might explain why some patients with COPD exacerbate more frequently than others. COPD patients with frequent exacerbations showed lower numbers of CD4+ central memory T-cells and CD8+ activated effector memory T-cells in peripheral blood when compared with patients that have infrequent exacerbations. This therefore provides a biological basis (or ‘endotype’) for the exacerbation susceptibility phenotype in COPD and suggests the presence of specific immune ‘signatures’ that may associate with exacerbation susceptibility.

We found a lower frequency and absolute number of central memory CD4+ T-cells in COPD patients who exacerbate frequently. Central memory cells are very sensitive to cross-linking of their T-cell receptors and rapidly express CD40 ligand in response [18]. Therefore, they are very easily and quickly activated in response to stimulation such that the immune system can respond more rapidly and effectively to previously encountered pathogens. Several studies have shown that CD4+ T memory cells contribute to an effective defence against specific viral pathogens (e.g. RSV and influenza) [19, 20]. Central memory CD4+ T-cells are stable and can maintain their population for many years [21]. This enables CD4+ Tcm to provide long-term protection against previously encountered pathogens. The lower frequency of CD4+ Tcm cells we observed in this study may predispose patients to viral respiratory infections and therefore to frequent exacerbations.

We hypothesise that the lower numbers CD4+ Tcm cells might be caused by chronic antigen stimulation in the frequent exacerbator phenotype, because long-term T-cell memory (i.e. Tcm cells) fails to develop in conditions of chronic antigenic stimulation. Numerous studies [22–28] have found that infections with high loads of chronically persisting antigen are characterised by sustained increased frequencies of effector cells. However, these cells fail to acquire essential features of memory cells, such as the IL-7 receptor; true for both CD4+ and CD8+ populations. Chronic antigen stimulation in frequent exacerbators may arise through multiple mechanisms, for example the past history of repeated infections, or exposure to alterations in the airway microbiome. It has long been recognised, for example, that the presence of potentially-pathogenic airway bacteria on sputum culture associates with susceptibility to exacerbation [16].

We also report a lower percentage of activated CD8+ effector cells in patients with frequent exacerbations. This finding might also be linked to chronic antigen stimulation, causing a downregulation of CD247 expression on CD8+ cells [29]. This process might be in part driven by myeloid derived suppressor cells (MDSC). Downregulating CD247 expression by MDCSs leads to an immunosuppressive state and defective effector cell function [29]. In support of this, both a downregulation of CD247 expression in pulmonary CD8 cells in COPD as well as higher levels of MDSCs have been seen in patients with COPD [8].

That we did not report differences in exhausted effector T-cells expressing PD-1 and regulatory T-cells might at first appear contrary to this hypothesis. Previous work by Kalathil did show differences in these type of immune cells and inferred that this might render COPD patients more susceptible to infections [8]. However, our findings suggest that the effect of exhausted T-cells and regulatory T-cells on exacerbation susceptibility may be limited. An alternative hypothesis would be that the PD-1+ T-cells and T-regulatory cells are indeed different in COPD patients compared to controls, but in the COPD population not specifically different in the frequent exacerbator phenotype compared to those less susceptible to exacerbations. We hypothesise that continuous lung damage due to smoking skews the systemic immune system in COPD patients towards an exhausted paralytic state as seen in studies by others [8, 9]. These changes in immune function lead to inadequate clearing of pathogens, and hence persistent chronic antigen stimulation. This persistent antigenic simulation, in return, directs the immune system in a subgroup of ‘frequent exacerbator’ COPD patients towards the changes seen in the present study.

In addition, the current study defines no differences within the systemic innate immunity (e.g. NK, iNKT, and γδ T cells) between FE and IE COPD patients. The explanation to the lack of differences might originate from the primary function of the innate immunity, which is predominantly to provide an initial defence against pathogens. Considering we studied COPD patients in a stable state (i.e. no acute infection), the innate immunity is not stimulated in the systemic circulation and differences cannot be observed. It might prove to be interesting investigating whether differences in the innate immunity arise between FE and IE during an acute COPD exacerbation.

Our findings are in agreement with the hypothesis that lung inflammation in COPD may lead to impaired immunity to respiratory pathogens, facilitating COPD exacerbations [8]. Both local and systemic inflammatory processes must balance between attenuating inflammation caused by smoking and launching an effective immunological response against pathogens. Our data suggest that in COPD patients susceptible to frequent exacerbations this balance is, at least in part, tipped towards attenuating the inflammatory response. It is tempting to speculate that restoration of immune function could restore this balance and might be of therapeutic benefit in COPD [7, 8].

Our higher dimensional statistical analysis did not show any single principal component responsible for most of the variance seen in our study. This highlights the heterogeneity associated with COPD. Whilst this might suggest that our findings therefore simply reflect type 1 errors, we think our findings truly reflects a systemic immune dysfunction because the direction of our results are in agreement with other studies on immune dysfunction in COPD [7, 8, 30]. However, the high heterogeneity in our sample, important in itself, emphasises that besides systemic immune dysfunction other mechanisms also likely play a role in the susceptibility to exacerbations [31].

A limitation of our study is the relatively small sample size and cross-sectional design, making it difficult to make firm causal inferences. Another limitation is that we did not perform functional analysis of the immune cell subsets, such as cytokine response when stimulated with pathogen exposure (e.g. RSV, influenza). Such a functional analysis might have revealed differences between FE and IE which would expand our understanding of the immune response in the exacerbation phenotype. A strength of our study is the detailed flow cytometry, the most comprehensive examination of various subsets of the adaptive systemic immune function ever reported in COPD. Moreover, it is one of the first studies to establish systemic immunological differences between phenotypes of COPD patients with regard to exacerbation susceptibility. Future prospective studies in a larger COPD population with frequent exacerbators should be undertaken to confirm our results.

## Conclusion

In conclusion, COPD patients who are subject to frequent exacerbations have measurable differences in the systemic adaptive immune system, which may make them more susceptible to exacerbations. Therefore, the exacerbation susceptibility phenotype in COPD has, at least in part, a biological basis, which can be detected with specific immune signatures in peripheral blood.

## Abbreviations

CD: Cluster of differentiation
COPD: Chronic obstructive pulmonary disease
FACS: Fluorescence-activated cell sorting
FE: Frequent exacerbator
FEV_1_: Forced expiratory volume in 1 s
FEV1%pred: Percentage of predicted forced expiratory volume in 1 s
FEV_1_/FVC: Ratio of forced expiratory volume in 1 s and forced vital capacity
FVC: Forced vital capacity
FVC%pred: Percentage of predicted
HLA-DR: Human leukocyte antigen - antigen D related
IE: Infrequent exacerbator
IL: Interleukin
iNKT: Invariant natural killer T cell
MDSC: Myeloid derived suppressor cells
NK: Natural killer cells
PBMC: Peripheral blood mononuclear cells
PC: Principal component
PCA: Principal component analysis
PD-1+: Programmed cell death protein 1
SORP: Special order research product
Tcm: Central memory T cell
Tem: Effector memory T cell
Tend: End-stage T cell
Treg: Regulatory T cell
